# Delivering early care in diabetes evaluation (DECIDE): a protocol for a randomised controlled trial to assess hospital versus home management at diagnosis in childhood diabetes

**DOI:** 10.1186/1471-2431-11-7

**Published:** 2011-01-19

**Authors:** Julia K Townson, John W Gregory, David Cohen, Sue Channon, Nicola Harman, Justin H Davies, Justin Warner, Nicola Trevelyan, Rebecca Playle, Michael Robling, Kerenza Hood, Lesley Lowes

**Affiliations:** 1 South East Wales Trials Unit (SEWTU), Department of Primary Care & Public Health, School of Medicine, Cardiff University, 7th floor Neuadd Meirionnydd, Heath Park, Cardiff, CF14 4YS, UK; 2 Department of Child Health, School of Medicine, Cardiff University, Heath Park, Cardiff, CF14 4XN, UK; 3 Health Economics and Policy Research Unit, University of Glamorgan, Pontypridd, CF37 1DL, UK; 4 Paediatric Psychology Department, Children's Centre, St David's Hospital, Cardiff, CF11 9XB, UK; 5 Medicines for Children Research Network Clinical Trials Unit, University of Liverpool, Liverpool, L12 2AP, UK; 6 Child Health Directorate, Southampton University Hospital Trust, Tremona Road, Southampton, SO16 6YD, UK; 7 University Hospital of Wales, Heath Park, Cardiff, CF14 4XN, UK; 8 School of Nursing and Midwifery Studies, Cardiff University, Cardiff, CF24 0AB, UK

## Abstract

**Background:**

There is increased incidence of new cases of type 1 diabetes in children younger than 15 years. The debate concerning where best to manage newly diagnosed children continues. Some units routinely admit children to hospital whilst others routinely manage children at home. A Cochrane review identified the need for a large well-designed randomised controlled trial to investigate any significant differences in comprehensive short and long-term outcomes between the two approaches. The DECIDE study will address these knowledge gaps, providing high quality evidence to inform national and international policy and practice.

**Methods/Design:**

This is a multi-centre randomised controlled trial across eight UK paediatric diabetes centres. The study aims to recruit 240 children newly diagnosed with type 1 diabetes and their parents/carers. Eligible patients (aged 0-17 years) will be remotely randomised to either 'hospital' or 'home' management. Parents/carers of patients will also be recruited. Nursing management of participants and data collection will be co-ordinated by a project nurse at each centre. Data will be collected for 24 months after diagnosis; at follow up appointments at 3, 12 and 24 months and every 3-4 months at routine clinic visits.

The primary outcome measure is patients' glycosylated haemoglobin (HbA1c) at 24 months after diagnosis. Additional measurements of HbA1c will be made at diagnosis and 3 and 12 months later. HbA1c concentrations will be analysed at a central laboratory.

Secondary outcome measures include length of stay at diagnosis, growth, adverse events, quality of life, anxiety, coping with diabetes, diabetes knowledge, home/clinic visits, self-care activity, satisfaction and time off school/work. Questionnaires will be sent to participants at 1, 12 and 24 months and will include a questionnaire, developed and validated to measure impact of the diagnosis on social activity and independence. Additional qualitative outcome measures include the experience of both approaches by a subgroup of participants (n = 30) and health professionals. Total health service costs will be evaluated. A cost effectiveness analysis will assess direct and indirect health service costs against the primary outcome (HbA1c).

**Discussion:**

This will be the first randomised controlled trial to evaluate hospital and home management of children newly diagnosed with type 1 diabetes and the findings should provide important evidence to inform practice and national guidelines.

**Trial registration number:**

ISRCTN: ISRCTN78114042

## Background

Across Europe, the incidence of type 1 diabetes in children younger than 15 years is predicted to rise by 70% between 2005 and 2020[1]. In some areas of the UK, between 1999 and 2003, the incident rate of newly diagnosed cases ranged from 22.4 - 29.8 per 100,000[1]. Traditionally, most children diagnosed with diabetes have been admitted to hospital as part of their initial management but over recent years there has been a move towards carrying out initial care from diagnosis in the home.

How children should be managed when newly diagnosed with diabetes is still strongly debated. Some units routinely admit all children to hospital, whilst others routinely manage children at home [2,3]. For some children, it is necessary to admit them to hospital due to clinical presentation, for example, for intravenous therapy if acidotic, (approximately 25% of children are acidotic at diagnosis). However, if children are not acutely ill at diagnosis, they can be managed safely in the community [3,4].

There is no high quality evidence concerning whether hospital admission or home management from diagnosis in children who are clinically well is different in terms of physical, psychological, social, and economic outcomes [5,6]. Indeed, a recent Cochrane Review[7] of this topic could draw no conclusions due to the very small number and low quality, or limited applicability, of studies. Although home management is supported as a safe, effective alternative to hospitalisation [3,8-10] studies have commonly been retrospective [5] with little account taken of any biases that may affect outcomes [3], and based on relatively small samples often from single centres [6].

There are also differing interpretations of home management, ranging from complete avoidance of hospitalisation [3] to 72 hours in hospital [8]. The study by Dougherty *et al *[8] was the only quality trial identified in the Cochrane review of home versus hospital management of type 1 diabetes in children [7] but did not strictly address the research question as children in the intervention group (n = 32) were also hospitalised for a total of 70 days. Studies examining cost effectiveness, mainly in the USA and Canada, suggest either cost reduction from outpatient management or no significant difference in costs between home and hospital management [8] but other outcomes need to be taken into account. For example, if either arm is found to reduce subsequent readmissions and result in improved glycaemic control, if sustained, this will have positive implications in relation to the reduced risk of diabetes related complications in later life[11]. These issues need to be examined over time, not merely to assess cost effectiveness but, more importantly, to determine the effect of home management and hospitalisation on patients' long term health and well being.

A recent empirical qualitative study [4] explored parents' experience of having their child managed at home from diagnosis and identified that, although parents experienced an initial grief response to the diagnosis similar to that usually associated with bereavement [12], they had a positive experience of home management. Parents believed that, because home management allowed them to deal with situations that occurred within the framework of their everyday life, the relative normality of this helped them feel more 'in control' of the situation to enable them to cope more effectively and feel less anxious. Surprisingly, although parents of children hospitalised at diagnosis have been found to experience, for example, remarkably high rates of post-traumatic stress symptoms [13] or distress due to their child's hospitalisation [14], no work has explored parents' experiences of initial hospitalisation or children's experiences of either approach.

Furthermore, little emphasis has been placed on psychosocial outcomes [6] or comparison of psychological outcomes from home management and hospitalisation. Improved pyschological well-being of parents and their affected child from diagnosis could also have a positive impact on diabetes control and subsequent engagement with the diabetes team. As Clar *et al *[7] emphasised, there is a need for a large, well- designed RCT to investigate whether there are significant differences in comprehensive short and long-term outcomes between the two approaches. The DECIDE multi-centred RCT will address these gaps in the current knowledge base by building on the programme of work by Lowes *et al *[2,11,12], providing high quality evidence on which to base decisions about the environment (home or hospital) where treatment should be initiated for children with newly diagnosed type 1 diabetes, potentially making a difference to the lives of children with type 1 diabetes and their parents.

## Methods/Design

### Ethical and governance approval

Multi-centre approval has been granted by Research Ethics Committee for Wales (**07/MRE09/59**). Site-specific approval has been granted by local RECs at all trial sites and all participating Acute Trust Research and Development Departments.

### Design

This is a multi-centre randomised controlled trial, for children aged 0-17 years who have been newly diagnosed with type 1 diabetes and their parents. Participants will be randomised to receive either hospital management (minimum of 3 overnight stays in hospital) or home management (no overnight stays in hospital).

### Clinic and patient selection

This study will be carried out in eight UK paediatric diabetes centres. Criteria for selection are based on size of the paediatric diabetes centres (a minimum of approximately 30/40 newly diagnosed patients per year) and geographical placement, to ensure balanced distribution of socio-economic factors across the UK. The paediatric diabetes centres will be based within NHS secondary care (paediatric wards and outpatient clinics). The paediatric diabetes team at each centre will comprise at least one consultant paediatrician with an interest in diabetes, a paediatric diabetes nurse and a paediatric dietician. Collaboration and recruitment is already agreed with teams in the UK from Cardiff, Southampton, Hull, Liverpool, Cambridge, Belfast, Newcastle and Nottingham.

Within each centre, a study-specific Project Nurse will be employed, whose role will be to co-ordinate nursing management of participants and data collection.

### Inclusion and exclusion criteria

Participants will only be entered into the trial if they meet the following inclusion and exclusion criteria (see Table 1)

**Table 1:** Participant inclusion/exclusion criteria

Inclusion criteria (Children)	Exclusion criteria (Children)
1. Children aged 0 - 17 years old	1. Children with ketoacidosis at presentation requiring treatment with intravenous insulin and fluids

2. Newly diagnosed type 1 diabetes (using recognised standard diagnostic criteria) who are clinically well at presentation	2. Children with a co-existing chronic disorder (e.g. cystic fibrosis) which will impact significantly on blood glucose control

3. Written informed consent given by child and assent from child	3. Children with Type 2 diabetes

4. Able to fill out study material (children aged ≥8 years old	4. Children with Maturity Onset Diabetes of the Young (MODY)

5. Written informed consent by parent(s)/carer	5. Children with an uncertain diagnosis

6. Parent/carer able to fill out study material	6. Children who are under the care of the local authority

	7. Children whose home circumstances are assessed as being unsuitable for home management

	8. Children who require hospitalisation for reasons other than their diagnosis

	9. Children who have a sibling with existing Type 1 diabetes

	10. Children who will begin treatment on a Continuous Subcutaneous Insulin Infusion (CSII)

### Recruitment

Patients (aged ≥8 years) and all parents/carers of patients (aged 0-17 years), will be given information about the study by a member of the clinical team to read whilst in the assessment unit/paediatric ward. They will have time to consider the study while blood tests are taken to confirm the clinical diagnosis.

### Randomisation

Once informed consent/assent is obtained, patients will be remotely randomised using an automated telephone system operational 24 hours a day. Patients will be randomised to either 'Home Management' or 'Hospital Management'.

Randomisation will be stratified by centre and balanced using randomly chosen permuted blocks. The randomisation ratio is 1:1

### Trial procedures

#### Process of care for all patients

Hospitalised and home-managed children and their parents will receive education and support from the project nurse and local paediatric diabetes team at each centre. Both cohorts will receive written information about diabetes, and a structured diabetes education programme. Education programmes, including dietary advice, will be standardized across centres as far as possible, but it is anticipated that minimal variability will be found concerning the educational content of programmes currently used at individual centres. Children will be commenced on an insulin regimen and delivery system that are deemed appropriate by teams at individual centres, and will be asked to undertake three to four blood glucose measurements a day before meals for the first 4 weeks of the study. All families will be given an appointment to attend the next appropriate diabetes clinic, will receive continued support from health professionals through telephone contact and clinic visits, and will be able to access help and advice out of office hours. Throughout the study, the paediatric diabetes team at each centre will comprise at least one consultant paediatrician, a paediatric diabetes nurse and a paediatric dietician.

To assist centres to deliver the study, a manual was developed providing guidance in key areas such as initial diagnosis, recruitment, home management and hospital management.

### Home management

The study will use a pragmatic approach to home management in order to accommodate the individual needs of participating centres. It would be inappropriate to undertake the study using one specific model that, if found to be beneficial, could not be subsequently adopted by centres due to, for example, differing insulin regimens (e.g. twice daily insulin or multiple injection regimens) or particular geographical needs (e.g. centres covering large rural areas).

The standard elements of home management that will be common to all participating centres are:

- Discharge home on the day of diagnosis with no overnight admission to hospital

- All treatment, education and support will be delivered at home or on an outpatient basis (attending ward/clinic for no longer than 2 hours for supervision of injections as necessary according to local need) for a minimum of three days (at least six supervised injections)

- Dietetic education will be provided at home or as outpatients, with continued dietetic support provided in clinic.

### Hospitalisation

Children will be admitted to hospital at diagnosis for a minimum of three nights (receiving at least six supervised injections while hospitalised). During their inpatient stay, families will receive treatment, education and support in the ward environment. The paediatric dietician will provide dietetic education to the child and family on the ward, and provide continued dietetic support at clinic visits. The project nurse at each centre will undertake at least one home visit (on or soon after the day of discharge at a time when insulin is due), with home visits continued as required according to the needs of individual families.

### Frequency & duration of follow-up

There will be three follow up appointments carried out at 3, 12 and 24 months as part of the participant's routine clinic visits.

Questionnaires will be sent to parents, and age-appropriate questionnaires to children, at 1, 12 and 24 month intervals.

### Data collection

Data concerning length of stay at diagnosis, glycaemic control (HbA1c), growth (weight, height, BMI), readmissions, adverse events (e.g. hypoglycaemia), home and clinic visits, school attendance, self-care activity and parents' time off work and travel costs (in relation to the child's diabetes) will be collected at clinic visits for 24 months after diagnosis. This will be at routine visits, which take place every 3-4 months.

There will also be standardised follow up visits at 3, 12 and 24 months. At diagnosis and at months 3, 12 and 24, extra blood from the initial sample taken to measure the patient HbA1c (glycosylated haemoglobin) level, will be taken and sent to a centralised laboratory (Diabetes Research Network Wales Laboratory, Llandough Hospital) for measurement of HbA1c concentrations. See Table 2.

**Table 2:** Data Collection

Month	Forms/samples
0	Diagnosis, consent and randomisation
	
	Baseline case report form (CRF)
	Blood sample - HbA1c analysis at central laboratory

1	Parent Questionnaire
	Child Questionnaire

3	Blood sample - for HbA1c analysis at central laboratory
	Follow up CRF

12	Blood sample - for HbA1c analysis at central laboratory
	Follow up CRF
	Parent Questionnaire
	Child Questionnaire

24	Blood sample - for HbA1c analysis at central laboratory
	Follow up CRF
	Parent Questionnaire
	Child Questionnaire

Throughout 24 month follow up	CRF completion and data collection at routine follow up visits approximately every 3-4 months.

up to 60 months	HbA1c measurements
	(taken and analysed at local Hospital)

### Primary & secondary outcomes

The primary outcome measure for the trial is HbA1c of patients at two years following diagnosis. HbA1c was selected because it is an objective measure of glycaemic control used to inform clinical practice and national and international policies and guidelines.

The secondary outcome measures for patients include growth, adverse events, psychological assessment of quality of life, coping with diabetes, diabetes knowledge, satisfaction and time off school. Secondary outcome measures for parents include anxiety, coping with diabetes, diabetes knowledge, satisfaction and time off work. Mean HbA1c at 3 and 12 months will be used to assess shorter term intervention effects. Additional qualitative outcome measures will be taken from health professionals' experience of both approaches to care. Finally, total health service costs, including hospitalisation, home/clinic visits and use of other NHS resources will be evaluated.

Quality of life measures to be used will be adapted from:-

- Issues in Coping with IDDM Scale - parent version[15]

- Spielburger Anxiety Scale (short version)[16]

- Diabetes Knowledge Scale - parent version [17]

- Quality of Life (PedsQol) - parent proxy version [18]

- Issues in Coping with IDDM Scale - child version[15]

- Diabetes Knowledge Scale - child version[17]

- Quality of Life (PedsQol) - child version[18]

In addition, there is no available validated measure of the impact of a diagnosis of diabetes on social activity and independence. Therefore, a Social Activity and Independence Questionnaire (SAIQ) will be developed and validated for use as a secondary outcome measure. The development phase will include item generation through interviews with children and parents living with diabetes and those items will be included for all participants as part of the main outcome questionnaires with parents and children.

### Sample size

In order for a randomised trial to have 80% power to detect an effect size of 0.4 (difference in mean HbA1c of 0.5% with an SD of 1.3% [7]) at a 5% significance level, a total of 200 patients would be required. To allow for loss to follow up of 17%, it is aimed to recruit 240 children. A previous study in Canada which evaluated reducing the amount of in-patient time at diagnosis showed a difference of 0.7% in mean HbA1c at two years[8]. Loss to follow up for the primary outcome of HbA1c should be small, as all of these patients will be attending clinic on a regular basis where HbA1c is monitored for clinical purposes.

### Analysis

#### Main analysis

Primary analysis will be intention to treat and will compare HbA1c between the two groups at the 24 month follow-up time point using ANCOVA. Baseline HbA1c will be included as a covariate. These analyses will be corrected for any clustering of outcomes within a clinic. Secondary outcomes analyses will compare the two groups using repeated measures ANOVA. These analyses will also be corrected for any clustering of outcomes within a clinic and baseline levels. Secondary analysis of the primary outcome using repeated measures ANOVA will be carried out using the 3, 12 and 24 month HbA1c values for the two groups and will also involve a more detailed exploratory analysis of the impact of clinic level factors on HbA1c outcome using a two level linear multi-level regression model.

#### Qualitative Analysis

Qualitative data analysis is an on-going rather than discrete activity. Data from the qualitative interviews undertaken with a subgroup of children older than 8 years of age and parents will be subject to thematic analysis, which comprises a number of steps: 1) interviews will be audio-recorded and transcribed verbatim, 2) patterns of experience will be listed, which may arise from direct quotes or the paraphrasing of common ideas, 3) data relating to the already classified patterns will be identified, 4) related patterns will be combined and catalogued into sub-themes, and 5) themes will be developed. Themes that emerge from participants' accounts will be coded and subsequently collated to form a comprehensive picture of their collective experience. It is anticipated that this will provide a detailed, in-depth insight into the thoughts and experiences of the participants. Data will be explored to allow identification and comparison of similarities and differences between cases and between the two arms of the study. The analytical process will be undertaken in a way that ensures that the integrity of the original document is kept intact.

#### Cost Effectiveness Analysis

A cost effectiveness analysis will assess direct (management at diagnosis) and indirect (subsequent) health service costs against the primary outcome (HBa1C). All NHS resource use, including inpatient admissions, insulin use, contacts with the diabetes team, investigations, attendances at accident and emergency departments, ambulance journeys, contacts with general practitioners and other health professionals will be monitored prospectively and valued by standard methods [19] using national unit costs supplemented where necessary by cost information from participating centres. Non-NHS costs such as parental time off work and travel are also being assessed but will be reported separately. Cost effectiveness results will be reported in the form of an incremental cost effectiveness ratio unless either form of patient management dominates (lower cost with greater effect). A series of one-way sensitivity analyses will test the sensitivity of the conclusions to changes in the main base case assumptions used.

Given the multiple objectives of a management at diagnosis service for children and the consequent importance of the secondary outcomes in this study (psychological adjustment, coping, adaptation to diagnosis), a costs-consequences analysis will also be undertaken.

## Discussion

The primary objective of this research is to determine whether there are significantly different outcomes for children with newly diagnosed type 1 diabetes who are clinically well, when they are either admitted to hospital or managed at home for initiation of insulin treatment and education of child and family.

This trial poses various challenges, one of which concerns the recruitment of patients. Recruitment takes place almost concurrently with the diagnosis and parents and children may find it difficult to think about participating in a study at this distressing time. It will be down to the skill and expertise of the DECIDE project nurses, and members of the multi-disciplinary paediatric diabetes teams, to explain the trial to potential participants, to maximise recruitment.

Another challenge of the trial is that many participating centres may not have had any previous experience of providing home management. This has necessitated the development of a dedicated DECIDE manual to assist centres through the process. This outlines for the first time, the minimum requirements of safe, home-based care from diagnosis which could be equally deliverable in a variety of differing clinical services.

This will be the first RCT to evaluate the difference, if any, in terms of clinical and psychological outcomes of patients managed from diagnosis in the differing settings. It is anticipated that outcomes from this trial will provide evidence to inform national and international guidelines (e.g. NICE, ISPAD) concerning how best to initially manage children diagnosed with type 1 diabetes.

## Competing interests

The authors declare that they have no competing interests.

## Authors' contributions

LL and JG are the joint principal investigators and guarantors of the study in its entirety. LL and JG were responsible for developing the research question and study design, and implementation of the study protocol. JT and NH were responsible for trial management. JT, LL and JG were responsible for drafting the manuscript. DC was responsible for designing the economic evaluation. SC was responsible for advising on study design and developing the SAIQ. JD, JW, MR and NT contributed to the study design and implementation.RP and KH were responsible for the statistical design and are the study statisticians. All those listed as authors were responsible for reading, commenting upon, and approving the final manuscript.

## Pre-publication history

The pre-publication history for this paper can be accessed here:

http://www.biomedcentral.com/1471-2431/11/7/prepub

## References

[B1] Patterson CC Dahlquist GG Gyurus MD Green A Soltesz G the EURODIAB Study Group Incidence trends for childhood type 1 diabetes in Europe during 1989 - 2003 and predicted new cases 2005 - 20: a multicentre prospective registration study The Lancet 2009 373 9680 2027 2033 10.1016/S0140-6736(09)60568-7 19481249

[B2] Lowes L Davis R Glasper EA, Lowson S Ambulatory care of children with newly diagnosed diabetes Innovations in Paediatric Ambulatory Care - A Nursing Perspective 1998 MacMillan Press Ltd, Hampshire UK 212 227

[B3] Swift PGF Hearnshaw JR Botha JL Wright G Raymond NT Jamieson KF A decade of diabetes: keeping children out of hospital British Medical Journal 1993 307 96 98 10.1136/bmj.307.6896.96 8343736 PMC1697706

[B4] Lowes L Lyne P Gregory JW Childhood diabetes: parents' experience of home management and the first year following diagnosis Diabetic Medicine 2004 21 531 538 10.1111/j.1464-5491.2004.01193.x 15154935

[B5] Lowes L Gregory JW Management of newly diagnosed diabetes - home or hospital Archives of Disease in Childhood 2004 89 934 937 10.1136/adc.2003.035949 15383437 PMC1719673

[B6] Charron-Prochownik D Maihle T Siminerio L Songer T Outpatient versus inpatient care of children newly diagnosed with IDDM Diabetes Care 1997 20 4 657 660 9096999 10.2337/diacare.20.4.657

[B7] Clar C Waugh N Thomas S Routine hospital admission versus out-patient or home care in children at diagnosis of type 1 diabetes mellitus Cochrane Database of Systematic Reviews 2007 2003 2 CD004099 10.1002/14651858.CD004099 12918002

[B8] Dougherty GE Soderstrom L Schiffrin A An economic evaluation of home care for children with newly diagnosed diabetes. Results from a randomised control trial Medical Care 1998 36 4 586 598 10.1097/00005650-199804000-00014 9544598

[B9] Chase HP Crews KR Garg S Crews MJ Cruikshanks KJ Klingensmith G Gay E Hamman RF Outpatient management vs in-hospital management of children with new-onset diabetes Clinical Pediatrics 1992 450 456 10.1177/000992289203100801 1643761

[B10] Lee PDK An outpatient-focused program for childhood diabetes: design, implementation and effectiveness Texas Medicine/The Journal 1992 88 7 64 68 1496490

[B11] Diabetes Control and Complications Trial Research Group The effect of intensive treatment of diabetes on the development and progression of long-term complications in insulin dependent diabetes mellitus The New England Journal of Medicine 1993 329 14 977 986 10.1056/NEJM199309303291401 8366922

[B12] Lowes L Gregory JW Lyne P Newly diagnosed childhood diabetes: a psychosocial transition for parents? Journal of Advanced Nursing 2005 50 3 253 261 10.1111/j.1365-2648.2005.03388.x 15811104

[B13] Landolt MA Ribi K Laimbacher J Vollrath M Gnehm HE Sennhauser FH Brief report: Posttraumatic Stress Disorder in parents of children with newly diagnosed type 1 diabetes Journal of Pediatric Psychology 2002 27 7 647 652 10.1093/jpepsy/27.7.647 12228336

[B14] Seppanen SM Kyngas HA Nikkonen MJ Coping and social support of parents with a diabetic child Nursing and Health Sciences 1999 1 63 70 10.1046/j.1442-2018.1999.00009.x 10894653

[B15] Kovacs M Iyengar S Goldston D Obrosky DS Stewart J Marsh J Psychological functioning among mothers of children with insulin dependent diabetes mellitus: a longitudinal study Journal of Consulting and Clinical Psychology 1990 58 2 189 195 10.1037/0022-006X.58.2.189 2335636

[B16] Marteau TM Bekker H The development of a six-item short form of the state scale of the Spielberger State-Trait Anxiety Inventory British Journal of Clinical Psychology 1992 31 301 306 1393159 10.1111/j.2044-8260.1992.tb00997.x

[B17] Beeney LJ Dunn SM Welch M Bradley C Measurement of diabetes knowledge - the development of the DKN scales Handbook of Psychology and Diabetes 1994 UK: Harwood 159 189

[B18] Varni JW Burwinkle TM Jacobs JR Gottschalk M Kaufman F Jones KL The PedsQL in type 1 and type 2 diabetes: reliability and validity of the Pediatric Quality of Life Inventory Generic Core Scales and type 1 Diabetes Module Diabetes Care 2003 26 3 631 7 10.2337/diacare.26.3.631 12610013

[B19] Drummond MF O'Brien B Stoddard GL Torrance GW Methods for the Economic Evaluation of Health Care Programmes 2005 3 Oxford: Oxford University Press

